# Assessment of the efficacy and safety of neuroendoscopic procedures for intracranial pathologies: A single-centre retrospective study with 318 intracranial endoscopic procedures

**DOI:** 10.1016/j.bas.2024.104142

**Published:** 2024-11-22

**Authors:** Mohammed Issa, Clara Dannehl, Carmen J. Büsken, Nieke Ueding, Angelika Seitz, Sandro M. Krieg, Andreas W. Unterberg, Ahmed El Damaty

**Affiliations:** a Department of Neurosurgery, Heidelberg University Hospital, Heidelberg, Germany; b Department of Neuroradiology, Heidelberg University Hospital, Heidelberg, Germany; c Faculty of Medicine, Heidelberg University, Heidelberg, Germany

**Keywords:** Neuroendoscopy, Intracranial, Adverse events and endoscopic third ventriculostomy

## Abstract

**Introduction:**

Neuroendoscopy has become a well-established procedure for treating various intracranial conditions.

**Research question:**

We evaluated the advantages of that technique, with focus on adverse events.

**Material and methods:**

Retrospective analysis included all patients who underwent neuroendoscopic procedures between January 2017 and December 2023. We conducted comparative analysis considering factors such as age, gender, follow-up duration, etiology, preoperative symptoms, clinical and radiological benefits, as well as surgical and nonsurgical adverse events rates.

**Results:**

Total of 318 neuroendoscopic procedures within 257 surgeries in 225 patients were included, with mean age of 18.8 ± 21.4 years. 170 cases (66.1%) were pediatric versus 87 cases adults (33.9%). Most common aetiologies were peri- and intraventricular cysts (27.2%), idiopathic aqueduct stenosis (24.9%), tumours (23.7%), and post-haemorrhagic hydrocephalus (17.1%). Procedures included endoscopic third ventriculostomy (51.0%), cyst fenestration (21.0%), and endoscopic-assisted ventricular catheter placements (19.5%). Headache was predominant preoperative symptom (42.0%). During follow-up, 84.8% and 82.5% of cases showed clinical and radiological improvement, respectively. Early surgical adverse events were observed in 5.4% of cases, while non-surgical adverse events occurred in 3.1%. There was no significant difference in the surgical adverse events rate between children and adults (4.7% vs. 6.9%, p = 0.563), compared to significant higher non-surgical adverse events in adults (6.9% vs 1.2%, **p=0.020**).

**Discussion and conclusion:**

Neuroendoscopy has demonstrated effectiveness and safety in treating intracranial diseases, boasting a low adverse events rate. Our study found no significant difference in the surgical adverse events rates between pediatric and adult groups.

## Highlights

• Neuroendoscopy has become a well-established procedure for treating various intracranial conditions.
• We evaluated the advantages of that technique, with focus on adverse events.
• Retrospective analysis included all patients who underwent neuroendoscopic procedures between January 2017 and December 2023.
• We conducted comparative analysis considering factors such as age, gender, follow-up duration, etiology, preoperative symptoms, clinical and radiological benefits, as well as surgical and nonsurgical adverse events rates.
• Total of 318 neuroendoscopic procedures within 257 surgeries in 225 patients were included, with mean age of 18.8 ± 21.4 years. 170 cases (66.1%) were pediatric versus 87 cases adults (33.9%). Most common aetiologies were peri- and intraventricular cysts (27.2%), idiopathic aqueduct stenosis (24.9%), tumours (23.7%), and post-haemorrhagic hydrocephalus (17.1%). Procedures included endoscopic third ventriculostomy (51.0%), cyst fenestration (21.0%), and endoscopic-assisted ventricular catheter placements (19.5%). Headache was predominant preoperative symptom (42.0%).
• During follow-up, 84.8% and 82.5% of cases showed clinical and radiological improvement, respectively. Early surgical adverse events were observed in 5.4% of cases, while non-surgical adverse events occurred in 3.1%.
• There was no significant difference in the surgical adverse events rate between children and adults (4.7% vs. 6.9%, p = 0.563), compared to significant higher non-surgical adverse events in adults (6.9% vs 1.2%, **p=0.020**).
• Neuroendoscopy has demonstrated effectiveness and safety in treating intracranial diseases, boasting a low adverse events rate.
• Our study found no significant difference in the surgical adverse events rates between pediatric and adult groups.

## 1 Introduction

Neuroendoscopy has emerged as the preferred surgical approach for various intracranial issues due to its effectiveness and minimal invasiveness. It is employed in managing peri- and intraventricular tumours, cysts, haemorrhages, infections, and occlusive hydrocephalus (Barber et al., 2013; G. Cinalli et al., 2005; Giuseppe Cinalli et al., 2007 et al.; M. A. Cinalli et al., 2023; Deopujari et al., 2023; Noris et al., 2023). Procedures include third ventriculostomy, tumour biopsy or resection, cyst fenestration, septostomy, haemorrhage evacuation, and ventricular lavage, often combining several interventions in one session. Notably, third ventriculostomy stands out as the most performed intraventricular endoscopic procedure (Deopujari et al., 2023; Di Rocco et al., 2006; El Damaty, Issa, Paggetti, Seitz and Unterberg, 2023; El Damaty, Marx, Fleck and Schroeder, 2017; Giannetti et al., 2015; Honeyman et al., 2022). Moreover, neuroendoscopy could assist in positioning the ventricular catheter during the implantation of a ventricular shunt system or external ventricular drainage. This method optimizes the placement of the ventricular catheter and yields superior outcomes compared to the freehand approach (Antes et al., 2017; Issa et al., 2023; Prajsnar-Borak et al., 2023; Roth and Constantini, 2012).

Despite significant advancements in neuroendoscopy technology and safety, clinical studies reveal complication rates ranging from 6.3% to 23.7%. Particularly, procedures involving intra- or paraventricular tumours exhibit the highest complication rates, followed by interventions through the aqueduct or involving the floor of the third ventricle including hypothalamus (Cerro Larrazabal et al., 2022; G. Cinalli et al., 2007a,b; Ebel et al., 2023; Peretta et al., 2006; Schroeder et al., 2004). Comparative analyses between pediatric and adult patients after such procedures indicate similar complication rates for both groups (Ebel et al., 2023; Girgis et al., 2015). These findings underscore the need for in-depth investigations to identify specific complications and prevent their recurrence (Chowdhry and Cohen, 2013).

The primary objective of this comprehensive study is to delve into the intricate landscape of morbidity and mortality rates linked to neuroendoscopic interventions. Spanning a substantial timeframe of 6 years from 2017 to 2023, this study presents a clinical series from a high flow neuroendoscopic center, describing the outcomes of 318 distinct endoscopic procedures administered via 257 surgeries in 225 patients. Retrospective data collection and statistical comparisons were performed between pediatric and adult patients.

## 2 Material and methods

Our single-center retrospective study encompassed individuals who underwent neuroendoscopic procedures for intra- and periventricular pathologies from January 2017 to December 2023. Adhering to the principles of the Declaration of Helsinki, the study received approval from the Ethics Committee of the University (Nr. S-084/2022). Informed consent was obtained from all patients or their parents/authorized caregivers. To evaluate the adequacy of the neuroendoscopy, brain Magnetic Resonance Imaging (MRI) was conducted before, after the surgical procedures and during follow-up for all patients.

A comprehensive analysis of various clinical parameters was conducted, encompassing factors such as age, gender, follow-up duration, pathology, operative technique, and preoperative symptoms. The study included the assessment of surgery-related and nonsurgical related complications, along with the recording of mortality rates.

Standard clinical and radiological follow-up examinations were routinely carried out before discharge, at 3 months post-surgery, and at the final follow-up, spanning a range of 6–81.3 months postoperatively. Standard brain MRIs were employed to assess the success of neuroendoscopic procedures.

We conducted thorough comparisons between pediatric and adult patients, delving into a detailed analysis of pathologies, surgical techniques employed, and the rates of complications encountered. This involved a meticulous examination of the specific nature of pathologies observed in each age group, the nuances of surgical approaches applied, and a comprehensive assessment of the varying rates of complications experienced within pediatric and adult populations. By investigating these aspects across different age categories, we aimed to discern any distinctive patterns or trends paving the way for more tailored and effective approaches to patient care.

### 2.1 Statistical analysis

We rigorously tested normal distribution using the Shapiro–Wilk test. Continuous variables were presented with mean ± standard deviation, while categorical variables were detailed with frequencies and percentages. Intergroup comparisons for continuous variables utilized the *t*-test, and for categorical variables, the Mann–Whitney-U and Fisher's exact tests were applied. A significance threshold of p < 0.05 indicated statistical significance. All analyses were conducted using SPSS 29 (IBM-Corp, Armonk, NY, USA).

## 3 Results

### 3.1 Patient's characteristics

In the study involving 318 neuroendoscopic procedures within 257 surgeries in 225 patients, the distribution of sex was nearly equal, with 132 males (51.4%) and 125 females (48.6%). The age of the participants ranged from 0.01 to 77.41 years, with an average of 18.8 ± 21.4 years. The follow-up period varied from 6 to 81.3 months, with an average of 29.3 ± 24.6 months. Among the cases, 170 (66.1%) were classified as pediatric cases. The surgical procedures had an average duration of 79.05 ± 49.5 min, and the ICU stay lasted for an average of 1.94 ± 5.5 days. Clinical improvement was noted generally in 84.8% of cases, and radiological improvement was observed in 82.5%. In around 165 surgical cases, cerebrospinal fluid (CSF) was intraoperatively obtained and subjected to examination. Generally, there was a marginal elevation observed in protein, lactate, and leukocyte levels beyond the norm. It is crucial to approach these results with caution, considering the diverse aetiologies involved. Table 1 provides comprehensive details on patient characteristics.

**Table 1:** patients’ characteristics.

Variable	Cases (%)
Patients	**225 (100)**
Cases	**257 (100)**
Sex	
Male	132 (51.4)
Female	125 (48.6)
Age^a^ in years	18.8 ± 21.4
Range	0.01–77.4
Follow-up^a^ in months	29.3 ± 23.4
Range	6.1–81.3
Pediatric cases	170 (66.1)
Surgery duration^a^ in minutes	79.1 ± 49.6
ICU stay in days	1.94 ± 5.5
Etiologies: rowhead	
Arachnoid and brain cysts	70 (27.2)
Idiopathic Aqueduct stenosis	64 (24.9)
Intraventricular Tumor	61 (23.7)
Posthemorrhagic hydrocephalus	44 (17.1)
Chiari malformation	11 (4.3)
Ventriculitis	7 (2.7)
Neuroendoscopic procedures:	318 (100)
Endoscopic third ventriculostomy (ETV)	131 (41.2)
Cysts fenestration	64 (20.1)
Ventricular catheter placement	62 (19.5)
tumor biopsy	21 (6.6)
Septostomy	18 (5.7)
Ventricular lavage	9 (2.8)
Endoscopic tumor resection	6 (1.9)
Aqueductoplasty	6 (1.9)
Choroid plexus cauterization (CPC)	1 (0.3)
CSF examination, content in average (Reference) rowhead	
Protein (<0.4 g/l)	0.50 ± 1.1
Glucose (49–75 mg/dl)	55.6 ± 14.6
Lactate (1.1–1.8 mmol/l)	2.1 ± 6.2
Leukocytes (<5/μl)	25.0 ± 163.3
Surgical complications	14 (5.4%)
Non-surgical complications	8 (3.1)
Clinical improvement	218 (84.8)
Radiological improvement	212 (82.5)
Postoperative hygroma	25 (9.7)

(%) Data in parenthesis are percentages.

a Data are given as mean ± standard deviation.

### 3.2 Aetiologies and procedures

The aetiologies of the cases included arachnoid and ependymal cysts (27.2%), idiopathic aqueduct stenosis (24.9%), intraventricular tumours (23.7%), and posthemorrhagic hydrocephalus (17.1%). Other aetiologies comprised Chiari malformation type 2 (4.3%) and ventriculitis (2.7%).

**Fig. 1:**
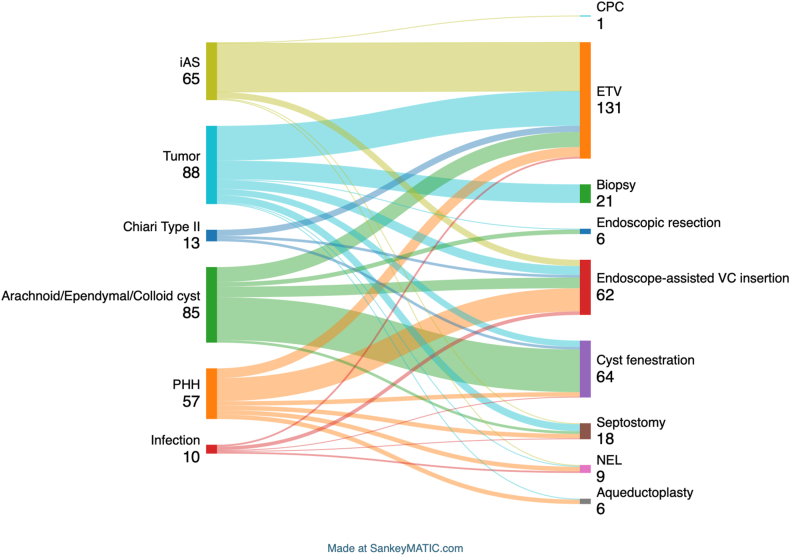
Sankey diagram showing distribution of indications and aetiologies of interventions of neuroendoscopic procedures.

Various neuroendoscopic procedures were conducted, encompassing a range of interventions. These included endoscopic third ventriculostomy (ETV) at a frequency of 41.2%, cysts fenestration (cystocisternostomy or cystoventriculosotmy) at 20.1%, and endoscope-assisted ventricular catheter placement at 19.5%. Additionally, tumour biopsy accounted for 6.6%, while endoscope tumour resection individually constituted 1.9%. Neuroendoscopic lavage (NEL) was performed in 2.8% of cases. Other procedures, representing 7.9%, involved aqueductoplasty, Septostomy, and choroid plexus cauterization (CPC). Fig. 1 depicts the distribution of indications and aetiologies of interventions within neuroendoscopic procedures.

### 3.3 Surgical adverse events and postoperative hygroma

**Fig. 2:**
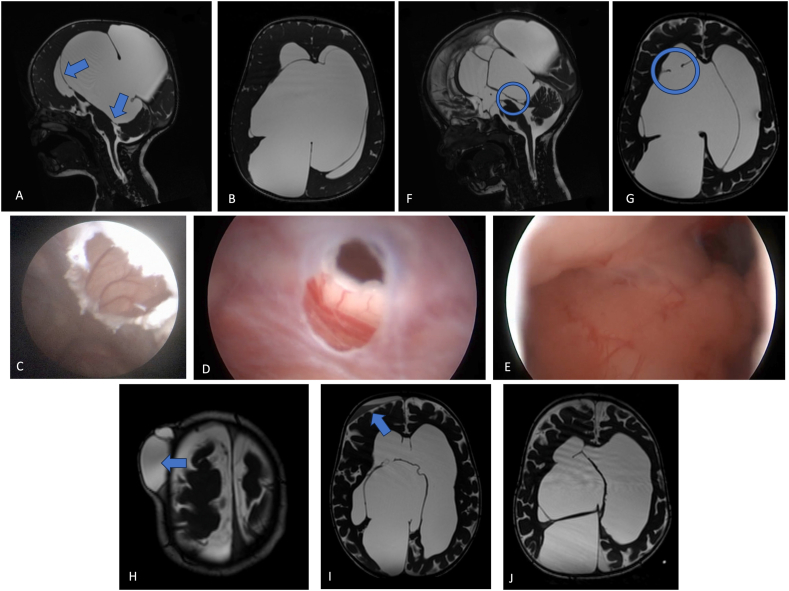
A: A sagittal CISS MRI of a 10-months old child who presented with macrocephaly, MRI showed a huge quadrigeminal arachnoid cyst compressing the whole ventricular system and nearby structures causing occlusive hydrocephalus, the blue arrows show the site of the planned fenestration to right lateral ventricle and 4th ventricle as aqueduct stenosis could not be excluded from preop. MRI. B: an axial CISS MRI showing the massive space occupying effect of the cyst exerted on the ventricular system. C: Intraoperative image showing the fenestration to right lateral ventricle. D: Intraoperative image showing the view inside the fenestrated 4th ventricle. E: Intraoperative image showing the fenestration through the roof of 4th ventricle and showing Foramen of Magendie. F: Postoperative CISS MRI showing the open aqueduct after release of pressure exerted from dorsally. G: An axial CISS MRI showing the performed fenestration to the right lateral ventricle. H: An axial CISS MRI showing the CSF collection subcutaneous at the site of the bur-hole. I: An axial CISS MRI showing the subdural hematoma. J: An axial CISS MRI 2 years after surgery resolution of CSF subcutaneous and subdural collections without new surgical interventions.

Adverse events were identified in 14 cases (5.4%) and underwent surgical revision. Hence, we classified them as surgical adverse events. Among them, two patients exhibited acute bleeding within the tumour mass following neuroendoscopic tumour biopsy and endoscopic third ventriculostomy (ETV). Wound healing issues manifested in three patients postoperatively. Four cases were associated with symptomatic cerebrospinal fluid leakage after a single ETV or ETV coupled with a tumour biopsy, necessitating reoperation and shunt implantation. Another patient developed hydrocephalus in alignment with the original etiology after endoscopic-tumour (Colloid cyst) resection and subsequently received a shunt implantation. One patient developed a subdural empyema following ETV and underwent a microsurgical evacuation via craniotomy. In a unique case, clamping the head in the Mayfield clamp resulted in two impression fractures in the skull cap, without a revision. Furthermore, postoperative hygroma was documented in 25 cases (9.7%). Two patients experienced postoperative bleeding, one following trauma and the other spontaneously. All patients successfully recovered without experiencing any neurological deficiencies. CSF collections or subdural hygromas which did not necessitate surgical interventions and resolved spontaneously during follow-up were not considered as adverse events, for example see Fig. 2 and video 1.

Supplementary video related to this article can be found at https://doi.org/10.1016/j.bas.2024.104142

The following is/are the supplementary data related to this article:

Multimedia component 1 Multimedia component 1

All goals were achieved in all surgeries, we did not experience surgeries which we needed to end due to technical difficulties or failure. Nevertheless, we tried to analyse the correlation between endoscopic procedure and surgical adverse events. As expected, tumor resections were the riskiest procedures with adverse events in 1/6 patients (16.7%) followed by biopsies where bleeding occurred in 3/21 (14%) of the biopsy procedures, followed by ETV in 5% and cyst fenestration in 3%. However, these adverse events rates were not statistically significant (p = 0.2) (See Table 3).

### 3.4 Non-surgical adverse events and mortality rate

Adverse events not necessitating surgical intervention were classified as non-surgical adverse events and manifested in 8 cases (3.1%). Among them, five patients experienced a postoperative cerebrospinal fluid (CSF) infection characterized by a combination of headache, prolonged fever, nuchal rigidity, elevation of C-reactive protein, CSF pleocytosis and positive CSF culture. This condition was successfully managed with antibiotic treatment. Two other patients developed postoperative epilepsy accompanied by hypoactive delirium and global cerebral diffusion disorders. Additionally, one patient who underwent endoscopic third ventriculostomy (ETV) exhibited temporary postoperative diabetes insipidus (See Table 3).

Tragically, four patients (1.56%) succumbed to their tumour disease, with a survival period ranging from 0.83 to 26.6 months after the surgery. No patient succumbed directly due to the surgical procedure.

### 3.5 Comparison between pediatric and adult patients

In terms of gender distribution, both groups exhibited a balanced representation, with no significant difference noted (p = 1.0). However, distinct variations emerged when considering age, with pediatric patients (below 18 years of age) showed a mean age of 5.4 ± 5.0 years, in contrast to the mean age of 45.1 ± 16.0 years observed in adult cohort (p < **0.001**). The follow-up duration was significantly longer by children (32.0 ± 23.4 vs 24.1 ± 22.6 months, **p = 0.01**), differences in surgical aspects became apparent. Pediatric cases had longer surgery durations (85.4 ± 52.2 min) compared to adult cases (66.7 ± 41.7 min) with a significant p-value of **0.002**. Similarly, pediatric patients experienced a longer stay in the ICU (2.34 ± 6.3 days) compared to their adult counterparts (1.14 ± 3.4 days), with a significant p-value of **0.036**. Examining the aetiologies revealed distinct patterns, with pediatric cases showing a higher prevalence of arachnoid and ependymal cysts, intraventricular tumours, idiopathic aqueduct stenosis, and posthemorrhagic hydrocephalus, all statistically significant (p < **0.001**). Additionally, the distribution of neuroendoscopic procedures varied between the two groups, where endoscopic third ventriculostomy (ETV), cysts fenestration and endoscope-assisted ventricular catheter placement were more frequently performed in pediatric cases (p < **0.001)**. Surgical adverse events showed no significant difference between the two groups (p = 0.562), while non-surgical adverse events were significantly higher in adults’ cases (p = **0.02**). Clinical improvement and radiological improvement were comparable between pediatric and adult patients, with p-values of 0.854 and 0.167, respectively. Notably, postoperative hygroma exhibited a trend toward significance, with a higher prevalence in pediatric cases (11.8%) compared to adult cases (5.7%) and a p-value of 0.181. Despite notable differences in aetiologies between the two groups, the examination of cerebrospinal fluid (CSF) revealed no significant distinctions in protein, Glucose, lactate, and leukocyte levels.

For an in-depth exploration of the comparison between adult and pediatric cases in the study, it is recommended to refer to Table 2.

**Table 2:** Comparison between pediatric and adult patients.

Variable Cases	Pediatric 170 (66.2)	Adults 87 (33.8)	p-value
Sex			
Male	88 (51.8)	44 (50.6)	1.0
Female	82 (48.2)	43 (49.4)	
Age^a^ in years	5.4 ± 5.0	45.1 ± 16.0	**<0.001**
Follow-up^a^ in months	32.0 ± 23.4	24.1 ± 22.6	**0.010**
Surgery duration^a^ in minutes	85 ± 52.2	66.7 ± 41.7	**0.002**
ICU stay^a^ in days	2.3 ± 6.3	1.1 ± 3.4	**0.048**
Etiologies:			
Arachnoid and brain cysts	48 (28.2)	22 (25.3)	
Posthemorrhagic hydrocephalus	42 (24.7)	2 (2.3)	
Intraventricular Tumor	33 (19.4)	28 (32.2)	
Idiopathic Aqueduct stenosis	33 (19.4)	31 (35.6)	**<0.001**
Chiari malformation	10 (5.9)	1 (1.2)	
Ventriculitis	4 (2.4)	3 (3.5)	
Neuroendoscopic procedures, n=301:	214 (67.3)	104 (33.7)	
Endoscopic third ventriculostomy (ETV)	72 (33.6)	59 (56.7)	
Ventricular catheter placement	55 (25.7)	7 (6.7)	
Cysts fenestration	45 (21.0)	19 (18.3)	
Septostomy	15 (7.0)	3 (2.9)	**<0.001**
Tumor biopsy	11 (5.1)	10 (9.6)	
Ventricular lavage	8 (3.7)	1 (1.0)	
Aqueductoplasty	5 (2.3)	1 (1.0)	
Endoscopic tumor resection	2 (0.9)	4 (3.9)	
Choroid plexus cauterization (CPC)	1 (0.5)	0 (0.0)	
CSF examination, content in average^a^ (Reference)			
Protein (<0.4 g/l)	0.57 ± 1.3	0.31 ± 0.42	0.054
Glucose (49–75 mg/dl)	54.1 ± 12.7	59.6 ± 18.1	0.058
Lactate (1.1–1.8 mmol/l)	1.82 ± 4.3	2.9 ± 9.6	0.468
Leukocytes (<5/μl)	29.2 ± 187.8	12.5 ± 33.1	0.353
Surgical complications	8 (4.7)	6 (6.9)	0.563
Non-surgical complications	2 (1.2)	6 (6.9)	**0.020**
Clinical improvement	145 (85.3)	73 (83.9)	0.854
Radiological improvement	136 (80.0)	76 (87.4)	0.167
Postoperative hygroma	20 (11.8)	5 (5.8)	0.181

(%) Data in parenthesis are percentages.

a Data are given as mean ± standard deviation.

**Table 3:** *Surgical and non-surgical adverse events* occurred in the early postoperative phase.

Surgical adverse events n = 14^a^ (5.4)	Frequency
CSF^b^ leakage	4 (28.6)
Wound healing issues	3 (21.4)
Postoperative bleeding (intraventricular, subdural or within the tumour mass)	4 (28.6)
Shunt implantation after endoscopic-tumour resection	1 (7.1)
Subdural empyema	1 (7.1)
An impression fracture in the skull vault due to head fixation in the Mayfield^c^	1 (7.1)

non-surgical adverse events n = 8^a^ (3.1%)	Frequency
CSF^b^ infection	5 (62.5)
Epilepsy	2 (25.0)
Diabetes insipidus	1 (12.5)

(%) Data in parenthesis are percentages.

a In 257 surgeries.

b Cerebrospinal fluid.

c Without need for revision surgery.

**Fig. 3:**
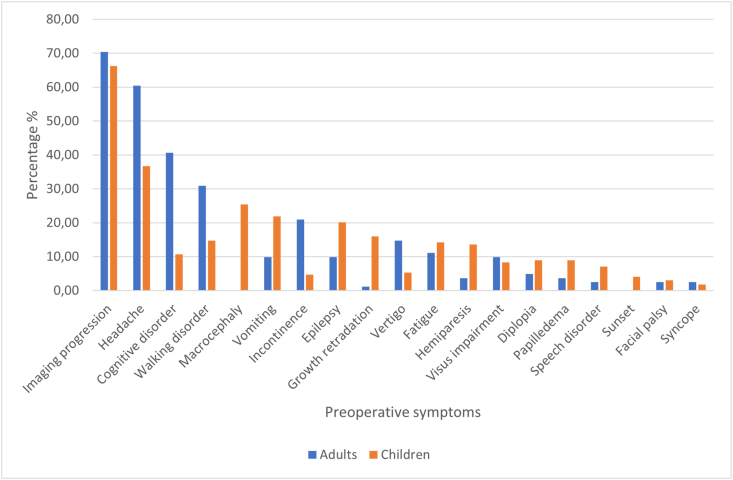
Preoperative symptoms' distribution observed in both children and adults.

The predominant preoperative symptom reported in both adults and children was headache, with macrocephaly being a prevalent accompanying symptom in children and cognitive disorders observed more frequently in adults. For further details on preoperative symptoms in both groups, refer to Fig. 3.

## 4 Discussion

Neuroendoscopy stands as a historical and well-established method for addressing intra- and periventricular pathologies. The continual evolution of technology and equipment has significantly mitigated adverse events, concurrently elevating the overall success rate of these procedures. Undoubtedly, the proficiency of the operator plays a pivotal role in achieving successful interventions, potentially contributing to a reduction in complication rates. Hence, meticulous documentation of cases becomes imperative to glean valuable insights (Chowdhry and Cohen, 2013; G. Cinalli et al., 2005; G. Cinalli et al., 2006; Ebel et al., 2023; Noris et al., 2023; Spennato et al., 2007; Vogel et al., 2011).

This study presents the most extensive single center series to date, encompassing 318 neuroendoscopic procedures in 257 surgeries, with a distinct emphasis on morbidity and mortality. Notably, our findings reveal an impressively low postoperative surgical adverse events rate of 5.4%, coupled with a non-surgical adverse events rate of 3.1%. These outcomes were observed over an average follow-up period of 29.3 months. Our results align with contemporary studies, underscoring the advancements in neuroendoscopic practices. In contrast, older studies portray a higher complication rate, emphasizing the progressive refinement and enhanced safety achieved in current neuroendoscopic procedures (Beems and Grotenhuis, 2004; Cerro Larrazabal et al., 2022; G. Cinalli et al., 2007a,b; Girgis et al., 2015; Peretta et al., 2006; Schroeder et al., 2004).

In 2007, Cinalli and colleagues published one of the most extensive series involving 231 intracranial neuroendoscopic procedures in children. Obstructive hydrocephalus emerged as the predominant etiology, accounting for 137 cases (59%). The complication rate stood at 13.8%, encompassing 32 cases. Among these, subdural hygroma manifested in 11 cases, with seven requiring subdural shunting. Cerebrospinal fluid (CSF) infection occurred in 11 cases, while CSF leak was observed in nine cases. Intraventricular haemorrhages were reported in two cases, technical failures in seven, subcutaneous CSF collection in one, and thalamic contusion alongside postoperative transient akinetic mutism in another case. In this study, there were no instances of patient mortality directly attributed to the procedure. One patient, accounting for a sudden death rate of 0.4%, succumbed six months post-procedure due to unexplained events. Additionally, three patients experienced enduring disabilities because of surgical complications, reflecting a permanent morbidity rate of 1.3% (G. Cinalli et al., 2007a,b). Conversely, our study demonstrated a comprehensive complication rate of 8.5%, comprising 5.4% required surgical revision and 3.1% which did not require surgical revision. Among these, 5 patients exhibited either cerebrospinal fluid (CSF) leak or postoperative hydrocephalus, necessitating treatment with a ventriculoperitoneal shunt. Hygroma formation was documented in 25 cases, while haemorrhage requiring evacuation developed in two instances. Notably, none of the hygroma cases required the implantation of a subdural-peritoneal shunt. Additionally, five patients developed postoperative CSF infection, managed successfully with antibiotics, and remained free from shunt-related interventions. Four patients (1.6%) in our study succumbed to their tumour-related diseases, ranging from one to 27 months post-surgery, independent of the endoscopic procedures. Importantly, none of the patients exhibited enduring neurological deficits. After analysing all surgical adverse events in our cohort and correlating them with type of endoscopic procedures aiming to achieve a risk stratification. We found that tumor resections were the riskiest procedures with surgical adverse events in 16.7%, followed by biopsies where bleeding occurred in 14.3%, ETV in 5.3% and cyst fenestration in 3.1%.

In 2023, Noris and colleagues conducted a current study involving 90 patients who underwent neuroendoscopic intervention for the treatment of loculated and multiloculated hydrocephalus. The study documented 17 surgical complications (18.9%), leading to the discontinuation of the operation in 9 cases due to bleeding or technical failure. Reoperation was required for three patients experiencing cerebrospinal fluid leaks, while two patients developed hygromas that were managed conservatively. Notably, our study did not encounter the need to interrupt any surgery due to intraoperative complications. However, postoperatively, two patients experienced bleeding in the biopsied tumour mass, necessitating revision.

In a study conducted in 2022, a total of 202 neuroendoscopic procedures were performed on 170 children, spanning from 2003 to September 2020. The predominant etiology of hydrocephalus was identified as intraventricular tumours (32.9%), followed by aqueduct stenosis (13.5%). The most frequently utilized procedure was third ventriculostomy, accounting for 62.9% of cases. Intraoperatively, 5 surgical complications were encountered, constituting a rate of 2.47% per procedure. In the postoperative period, systemic complications were noted in 23.7% of cases per procedure, with 12.87% being neurologic, 8.41% hormone-related, 10.9% fluid-related, 0.5% haemorrhagic, and 0.99% resulting in postoperative mortality (Cerro Larrazabal et al., 2022).

In the retrospective study conducted by Ebel and colleagues, the investigation into disparities in intracranial neuroendoscopy between children (n = 47, 35.6%) and adults (n = 85, 64.4%) revealed similar rates of both transient and permanent complications in both groups. However, a notable difference emerged in the revision rates, with children experiencing a higher rate at 38.3%, compared to adults with a revision rate of 17.6%. Tumour was identified as the most common indication in children, whereas adults predominantly presented with aqueduct stenosis (Ebel et al., 2023).

In our study, a parallel comparison between pediatric and adult patients was conducted. In this analysis, indications for intracranial neuroendoscopy were significantly different in both groups. However, the revision rate demonstrated similarity, standing at 4.7% in pediatric patients and 6.2% in adults. Interestingly, non-surgical adverse events were found to be significantly higher in adults, with a p-value of **0.015**. Both pediatric and adult groups exhibited favourable long-term results, showcasing clinical and radiological improvements akin to the findings in the previously mentioned study (Ebel et al., 2023). While we observed a potential increase in hygroma formation in children, the difference did not reach statistical significance.

### 4.1 Limitations

Caution is advised in interpreting the findings of this study, given its retrospective design and the relatively modest sample size of patients. We would like to emphasize the specific nature of complications, particularly focusing on bleeding, CSF infections and leakage. It's crucial to note that the documentation of neurocognitive long-term complications resulting from intracranial, especially intraventricular, neuroendoscopic interventions on the floor of the third ventricle was not included in this study. These aspects are well-documented in existing literature(Soleman and Guzman, 2020). However, recognizing the significance of this information, we stress the need for prospective long-term studies to thoroughly investigate and understand these aspects.

## 5 Conclusion

The efficacy and safety of neuroendoscopy in addressing intracranial diseases have been well-established, showcasing a commendably low rate of complications. Our study, in alignment with previous research, discerned no significant disparity in the occurrence of surgical adverse events when comparing pediatric and adult groups. This consistency in outcomes underscores the robustness and reliability of neuroendoscopic interventions across different age cohorts. The findings contribute to the growing body of evidence supporting the favourable risk profile associated with neuroendoscopic procedures in the management of intracranial conditions.

## Informed consent Statement

Written informed consent has been obtained from the patient(s) to publish this paper.

## Author Contributions

Conceptualization, MI and AeD.; methodology, MI and AeD; software, MI; validation, MI, AeD, AWU and SMK; formal analysis, MI; investigation, SMK; resources, AWU and SMK; data curation, CD, CJB, NU and MI; writing—original draft preparation, MI; writing—review and editing, AS, AWU, SMK and AeD; visualization, AS and AeD; supervision, AeD and SMK.; project administration, AeD; funding acquisition, AWU and SMK. All authors have read and agreed to the published version of the manuscript.

## Institutional review Board Statement

The study was conducted according to the guidelines of the Declaration of Helsinki and approved by the Institutional Ethics Committee of Heidelberg University (protocol code Nr. S-084/2022 and date of approval).

## Data Availability Statement

The data presented in this study are available on request from the corresponding author.

## Funding

This research received no external funding.

## Conflicts of interest

The authors declare no conflict of interest.
